# Being Sure and Living Well: How Security Affects Human Flourishing

**DOI:** 10.1007/s10790-021-09870-6

**Published:** 2022-01-03

**Authors:** J. A. M. Daemen

**Affiliations:** Institute of Political Science, Faculty of Social and Behavioural Sciences, Leiden University, Pieter de la Court Building, Wassenaarseweg 52, 2333 AK Leiden, The Netherlands

## Introduction

Why do we consider a worker with a permanent contract better off than one on temporary hire? To some extent, this has to do with the symbolic value of a permanent contract: it serves as a sign of recognition and trust on the part of the employer vis-à-vis the employee. But most importantly, we consider such a contract more valuable because it gives the worker more security. And security, we feel intuitively, is good for people. This is not only true for security of bodily integrity, that is, security in the narrow sense of the word. It applies to security of all things that contribute to a person’s well-being, such as her health, her income, or her relationship. But why exactly would *security* of something that contributes to a person’s well-being, also *itself* contribute to her well-being? Are there ways in which security of a good might actually be bad for someone? Just how much security should we aim to create for ourselves? These are the questions that concern me here.

In a way, these are old and familiar issues. It seems plausible that human beings have always found their well-being importantly to hinge on security of goods, be it at first perhaps only of food and the tools used to procure that food. Two great utilitarian thinkers already addressed the significance of security for people’s well-being back in the 19^th^ century. According to Jeremy Bentham (1843), “we must consider that man is not like the animals, limited to the present, whether as respects suffering or enjoyment; but that he is susceptible of pains and pleasure by anticipation; and that it is not enough to secure him from actual loss, but it is necessary also to guarantee him, as far as possible, against future loss” (p. 110). John Stuart Mill (1871) described security as “the most vital of all interests”, stating that “[w]e depend on it for all our immunity from evil” and “for the whole value of every single good that goes beyond the passing moment; because if we could be deprived of anything the next instant by whoever was at that moment stronger than ourselves, nothing could be of any worth to us except the gratification of the instant” (p. 81).

Questions about security and well-being are clearly relevant in today’s society as well. Around the world people currently seem concerned about security of many different kinds of goods. The corona pandemic compromises security of our health and that of our loved ones, flexibilisation of labour makes job and income security less accessible for a large part of the workforce, and climate change leads many to worry about their future living environment. Nonetheless, thinkers in contemporary analytic political philosophy have paid rather little attention to the topic of security. There are a couple of notable exceptions. Waldron (2003, 2006) has done useful exploratory groundwork on the concept and value of security. Wolff and De-Shalit (2007) have provided an illuminating account of the ways in which people are disadvantaged when their functionings are or become insecure. John (2011) has developed a stimulating argument for considering physical security a constituent of well-being. But the most important source to be acknowledged here is the work of Herington (2015, 2017, 2019). Particularly valuable is his analysis of the contribution that security makes to well-being, which he investigates from the perspectives of hedonic, desire-satisfaction, and objective-list theories of well-being (2019).^1^

This paper is an attempt to deepen our understanding of the ways in which security affects well-being. It adds something new to the existing literature in a number of ways. Firstly, it contributes to our knowledge of what security actually is. It builds on the conceptual framework of Herington (2019) by distinguishing between “fact-security”, “belief-security”, and “emotion-security”, but proposes a new set of definitions for these concepts (section 2). Secondly, different from the writings on the value of security mentioned above, this work relates security to the idea of human flourishing. This is a specific understanding of well-being (section 3). Thirdly, this study pays special attention to the importance of alignment between fact-security, belief-security, and emotion-security. When and why it matters whether the facts, our beliefs, and our emotions are in line with one another when it comes to security, becomes clear from the investigation of the ways in which security can make a positive contribution to our well-being (section 4). The last novelty of this paper lies in its exploration of the question whether security might also impact our flourishing negatively. It is demonstrated that there are indeed ways in which security can hamper our well-being sometimes (section 5). I conclude by reflecting on the question what we can learn from these findings in shaping our own lives and working towards a better society (section 6).

## Security as Sureness

What is security? At the most general level, security, as it is understood here, is a mode in which a *person* can relate to a *good* (see Herington 2017, p. 187; Waldron 2006, p. 462). But what exactly does this mode entail? In answering this question, it is common to distinguish between “objective” and “subjective” security, whereby the former refers to some fact about a person’s enjoyment of a good, and the latter concerns her own experience of the matter.

Herington (2019), however, replaces this distinction by a more refined conceptual framework comprising three separate concepts of security. Firstly, there is “fact-relative security”. According to Herington, “[t]he fact-relative security of some prudential good *G* for an individual *S* at some time *t* is the *objective* probability that *S* will enjoy *G*, given the state of the world at *t*” (p. 183). It is the answer to the question: what is the *actual* chance that this person will enjoy this good? Fact-relative security refers purely to the actual state of the world; what a person thinks or feels about this is irrelevant. Secondly, there is “belief-relative security”. On Herington’s definition, “[t]he belief-relative security of some good *G* for an individual *S* at some time *t* is *S*’s *subjective* probability of enjoying *G*, given her beliefs at *t*” (ibid.). It is the answer to the question: how likely does this person *herself* think it is that she will enjoy this good? Belief-relative security refers purely to the actual beliefs of a person; whether these are justified or true does not matter. Thirdly, there is “the affect of security” or “affective security”. On Herington’s view, “[t]he affect of security is an emotional state of calm assurance” (p. 184). It is not per definition experienced in regard to a good and it is not necessarily attached to a certain belief or state of the world; it is just a feeling.

The distinction between fact-relative, belief-relative, and affective security is of great value when we try to make sense of security and its effects on well-being. It allows us to acknowledge that in practice these three things can, and often do, come apart. Take climate change. Judging from the most recent scientific reports, it seems highly likely that the climate will change considerably even during the lives of people currently inhabiting the earth – meaning that they have only low fact-relative security of an unaltered climate. At the same time, quite a number of people seem to underestimate the risk of climate change – meaning that their belief-relative security of this good is far higher. Finally, the threat of climate change seems to emotionally upset people only from time to time – meaning that sometimes they do have affective security, and sometimes they do not. The distinction between the factual dimension, belief dimension, and emotional dimension of security will also prove to be helpful when we investigate the ways in which security affects human flourishing. Someone’s fact-relative security of a livable climate is beneficial for her in a different way than her belief-relative security of it, and fact-relative and belief-relative security in turn make a different contribution to her well-being than affective security. Yet, we will see that for some benefits of security to obtain fully, it is indeed important that the three match up.

Notwithstanding the great value of Herington’s conceptual framework, I want to put forward a different set of definitions that I think to be more suitable for the project of investigating how security affects well-being. In order to distinguish my definitions from Herington’s, I will keep referring to his definitions as “fact-relative security”, “belief-relative security”, and “affective security”, and propose my own under the simpler headings of “fact-security”, “belief-security”, and “emotion-security”. I would like to propose two modifications to Herington’s definitions. The first modification concerns fact-relative and belief-relative security. My proposal is to avoid the notion of probability that Herington uses in his definitions of fact-relative and belief-relative security, and to describe fact-security and belief-security rather in absolute terms. Whereas Herington’s concept of fact-relative security refers to the objective *probability* that a person will enjoy a good, my concept of fact-security refers to the flat-out *fact* that she is bound to enjoy this good.^2^ And whereas Herington’s concept of belief-relative security refers to the subjective *probability* that a person will enjoy a good, my concept of belief-security refers to her flat-out *belief* that she will enjoy this good.

In order to see why it would be better to avoid the notion of probability in the definitions of fact-security and belief-security, consider the following. In this research, we are interested in the effects of security on well-being. Therefore, it seems most logical to define security as a thing that can have effects. When we define security as the probability that a person will enjoy a good, however, security is not a thing that can have effects. After all, like concepts such as “height” or “weight”, the notion of “probability” does not *itself* refer to a particular condition. Rather, it is a scale, a parameter, on which many different scores are possible. Each such a *score* – 0%, 100%, or any score in between – *does* refer to a particular condition. But “probability” itself does not. Thus, if we are to understand security as *itself* as a particular condition that can have real effects that we can actually think through, we should not define it as probability. Instead, we should see it as the ideal found at the upper end of the probability scale. In the case of fact-security, this is the flat-out fact that a person is bound to enjoy a good. In the case of belief-security, this is her flat-out belief that she will enjoy this good.

This is not to say that fact-security and belief-security are all-or-nothing phenomena. Someone may very well have security of something *to a certain extent*. And the extent to which she has security can indeed be seen as a matter of probability. First, consider fact-security: the fact that a person is bound to enjoy a good. When indicating to what extent this obtains for her, the relevant probability is the objective probability that she will enjoy this good, i.e. the chance that would be calculated by an all-knowing observer.^3^ Second, consider belief-security: a person’s belief that she will enjoy a good. When indicating to what extent this obtains for her, the relevant probability is her subjective probability that she will enjoy this good, i.e. the chance estimated by herself.^4^ But even though the *extent* to which a person has fact-security and the *extent* to which she has belief-security can be considered *matters of* probability, this does not mean that fact-security and belief-security should *themselves* be *defined as* probabilities. As I argued above, I think they should not.

Herington’s definition of affective security does not suffer from the same problem as his definitions of fact-relative and belief-relative security. After all, his concept of affective security already refers not to a parameter but rather to a particular condition – namely a feeling of calm assurance. Still, I would like to propose a modification with respect to affective security as well. My proposal is to avoid the positive phrasing that Herington uses in his definition of affective security, and to describe emotion-security in negative terms instead. Rather than itself “a felt quality of tranquility” (Herington 2019, p. 184), I take emotion-security to be the absence of anxiety or fear.

The reason why I think this definition is better, is that what we generally describe as a feeling of “security” is not so much an emotional sensation that is directly noticeable itself. Rather, it is an emotional state that functions as a background against which we experience our more direct affects. Indeed, emotion-security is better defined not by what it is, but by what it is not: it is simply the absence of fear. Herington (2019) seems to have two reasons for describing affective security as itself a directly noticeable emotional sensation. Firstly, he mentions some examples in which people appear to experience security as such: the lost toddler who is reunited with her parents; the job seeker in a bad market who is offered work; the patient with a suspect mole who finds out it is benign (p. 184). However, the affect that he is alluding to in these situations seems more accurately characterised as feeling of relief than as one of security. Secondly, Herington refers to a number of historical writings in which security is defined positively, as a calm mental state. At the same time, as becomes clear from Herington’s own analysis of security in the history of political thought, it seems that it has even more often been interpreted negatively, as “freedom from fear” (Herington 2015, p. 25). If we are interested in the contribution that the feeling of security makes to well-being, we thus seem to be justified in taking it to consist in the absence of fear.

The proposed modifications give us the following conceptual framework: fact-security is the fact that a person is bound to enjoy a good; belief-security is a person’s belief that she will enjoy a good; and emotion-security is the absence of fear. Each of these three can obtain to a certain degree, but none are defined as a degree. As suggested before, in practice it is often the case that only one or two of these three obtain, or that each obtains to a different extent. Still, I think they can be viewed as three aspects of one ideal. This ideal I call “security as sureness”. If and only if a person has both fact-security *and* belief-security of a good *and* she experiences emotion-security, and thus she can be said to “be sure” in a factual sense *and* a belief sense *and* an emotional sense, does the ideal obtain in full. So, from my point of view, someone who is actually about to get a contract extension but is still ignorant of this fact and frets about losing her work cannot be said to have full security with regard to her job.

Some might think that it is wrong to incorporate not only a factual aspect but also a belief aspect and an emotional aspect into the concept of security. They might suggest that only fact-security is *actually* security, belief-security is just the *belief that one has* security, and emotion-security is merely the *feeling associated with* security. From this point of view, the person who is bound to have her contract extended but does not know this yet and feels anxious in this regard does indeed have security of her job; it is just a pity that she suffers from “pathological” beliefs and emotions. My hunch, however, is that most people would *not* consider this person as having full job security: the belief aspect and emotional aspect are too central to the notion of security to be disregarded like that. I therefore stick with my view that security only obtains fully if someone has belief-security and emotion-security as well as fact-security.

Security as sureness should be distinguished from a number of other concepts. First of all, it is different from continuity. Security is about a person’s enjoyment of a good in the future. It does not say anything about whether or not she already enjoys this good in the present. Consider the example of someone who signed a contract to start a job next month: even though she does not have the position at this point already, she does have security of it.^5^ Security is also not the same as unconditionality. A person can have security of a good – say, an income – even though she has to meet certain requirements in order to continue or come to enjoy this good – say, she has to work – provided that she is sure to meet these requirements.^6^ Furthermore, security differs from control. We can very well have security of things that are not under our control. The weather, for instance, does not depend on our will, and still we can be sure to have nice weather in the weekend.^7^ Security is also different from certainty. Someone can have or claim certainty of beliefs about various kinds of issues – from what is the speed of light to the reason why England lost the football cup final – but only certainty of beliefs about one’s future well-being are directly relevant for one’s security.^8^ Lastly, security should be distinguished from robustness. Robustness is about whether a person *would* enjoy a good – say, her friend’s support – if circumstances were relevantly different than the way they actually are – say, if she were chronically ill. Security, on the other hand, concerns whether someone *will* actually enjoy a good as time goes by.^9^ Of course, there are important connections between security and these other notions. Yet, security is a comprehensive and distinct concept worth studying on its own.

## Human flourishing

Before delving into the question of how security relates to well-being, let me explain how well-being is understood here. I adopt a concept of well-being that I refer to as “human flourishing”. This term is sometimes used as a translation of Aristotle’s (2000) notion of *eudaimonia* specifically. Here, however, it is used as an objective concept of well-being more generally. It points us to what things in life have prudential value, that is, what things are good for human beings.

If we take well-being to consist in human flourishing, we adopt an objectivist theory of the good. Such a theory can be contrasted with two subjectivist theories of the good: hedonism, which takes a person’s well-being to consist only in her experience of pleasure, and desire-fulfilment theory, which takes someone’s well-being to consist exclusively in the satisfaction of her desires. Instead of providing a full-blown defence of human flourishing as an understanding of the good, let me just highlight two important shortcomings in the other two theories, which we can overcome if we adopt an objective concept of well-being. Hedonism implies that nothing can matter prudentially to a person but the quality of her experience (Arneson 1999, p. 114). On this view, the life of a person who *really* makes a friend or writes a great novel is no better than the life of a person who has the mere *illusion* that she does so – the experience, after all, is the same for both (Nozick 1974, p. 42). Many people find this unintuitive and therefore renounce the hedonic understanding of well-being. Desire-fulfilment theory, at least on its simplest version, implies that the satisfaction of a person’s desires is good for her regardless of the content of these desires. On this view, fulfilment of a person’s desires is good for her even if these desires do not seem to regard her *own* life – think of a desire that distant strangers are adequately nourished – or if she has these desires only because she is *confused* – think of a desire to eat food that will actually make her sick (Arneson 1999, p. 124). Again, many people find this not intuitive and therefore renounce the idea of well-being as desire-fulfilment.

An objectivist theory of the good does not suffer from these difficulties, because it does not make a person’s well-being hinge entirely on her subjective experience or desires. Instead, it puts forward an objective list of the things that a good human life consists in – the things that make up human flourishing. Different philosophers have included different things in this list. Many of them thereby refer to the natural characteristics that humans have as the kind of beings they are. Kraut (2007), for example, takes human flourishing to consist in the possession, development, and enjoyment of our physical, cognitive, affective, sensory, and social powers (pp. 136–137). Others focus on the things people can obtain by putting these characteristics to use. Rasmussen (1999), for instance, mentions health, knowledge, achievement, pleasure, and friendship (p. 4). As these lists suggest, the experience of pleasure and the satisfaction of desires may very well contribute to people’s flourishing – it is just not exhaustively made up of these things.

I will not spell out what I take human flourishing to consist in specifically here. Instead of starting from a particular list of items and figuring out how security relates to each of these, I will try and make intuitive what a flourishing human life looks like and how security fits into this picture as we go. A couple of points, however, need to be clarified in advance. From the viewpoint of human flourishing, something can be good for someone for two different reasons: either it forms a *component of* a flourishing human life, or it is an *instrument for* leading such a life. In the first case, it has intrinsic value; in the second case, its value is instrumental. Whether one sees something as a component or rather as an instrument depends on the definition of human flourishing one adopts. On Kraut’s view, for instance, the use of my social skills would count as a component of my flourishing; Rasmussen, on the other hand, would see it an instrument for achieving a component of flourishing (friendship, for example).

It is important to note that, when we consider a person’s *security* of a *good*, there are two factors at issue that potentially affect this person’s flourishing. The first one is the good that this person has security of. Throughout this text, the noun “good” is taken to refer to something that contributes to this person’s well-being, in some way, at some point, either as a component of or an instrument for her flourishing. This definition is deliberately kept vague so that a variety of things that we might speak of as matters that we can have some degree of security of can fit under this heading – from concrete stuff like our bicycle or our skin, to more abstract goods such as our ability to move or our sensory pleasure. The second factor potentially affecting human flourishing that is implied when we consider a person’s security of a good, is security itself. As was explained above, security comprises a fact, a belief, and an emotional condition. The main question of this paper is whether and how these three things impact people’s well-being, be it as components of or instruments for human flourishing. Let us turn to that question now.

## What is good about security?

Why would it be good for a person to have security of a good? Remember that the ideal of security as sureness has three aspects: fact-security, belief-security, and emotion-security. Below, I investigate how each of these may contribute to human flourishing. I will thereby consider them separately, but also indicate when and why it is important that one aspect of security is backed up by another aspect of security in order for the benefits to obtain.

Firstly, consider fact-security. Recall that fact-security is the fact that a person is bound to enjoy a good. Also remember that the good security of which is at issue must be something that somehow, sometime, contributes to this person’s well-being, as a component of or an instrument for her flourishing. Let us take some nice house to live in as an example. When a person has fact-security of this house at some point, this entails that she will in principle continue or come to enjoy this house at a later point. Generally, her well-being will be positively affected by this *at that later point in time*. It must be noted, however, that it really is the house itself that contributes to this person’s flourishing, rather than her fact-security of it. Hence, the contribution only obtains when the house stays or becomes present in her life, not at the point she only has fact-security of it. Strictly speaking, a person’s fact-security of a good therefore does not itself contribute to her flourishing. In other words, the mere fact that we are bound to enjoy a good tomorrow, does not make us any better off today.^10^ Still, it is important to recognise the role of fact-security in our well-being over time: if we have fact-security of goods now, this generally means that we will flourish later. Indeed, it is this aspect of security that we normally consider most significant: we primarily care about security of, say, our house, not because of the contribution this security makes to our current well-being, but because of the contribution this house will make to our future well-being.

Secondly, consider belief-security. This was defined as a person’s belief that she will enjoy a good. Shortly, I will explain in what way belief-security can contribute to human flourishing. But first, let me consider and refute an argument for the value of belief-relative security developed by Herington (2019). He suggests that belief-relative security contributes to our well-being because we need it in order to make rational plans. Before I go on to challenge this view, a few words on the role planning can be seen to play in a flourishing life. As mentioned before, many philosophers take the possession and exercise of the core human properties to be crucial for human flourishing. From an Aristotelian viewpoint, one of the essential human properties is that of practical rationality (Hurka 1993). Making rational plans, in turn, is one of the central activities in which we put this property to practice. And for this activity, Herington (2019) argues, belief-relative security is critical.

The argument is as follows. For plans to be rational, they must be means-end coherent. Bratman (2009) explains it this way: “The following is always pro tanto irrational: intending E while believing that a necessary means to E is M and that M requires that one now intend M, and yet not now intending M” (p. 413). Now, Herington (2019) argues that “[i]n order to make means-end coherent plans, we must believe (or presuppose) that we will possess all of the necessary means to realizing those plans” (p. 198). He appeals to the example of someone who believes she will likely die from a congenital heart defect soon, and therefore is unable to make complex rational plans for the future. He concludes that we need belief-relative security, at least of our vital needs, in order to function as rational planners.

On closer inspection, however, this argument does not hold. Plans are different from fantasies in the sense that plans are actually intended to be carried out. Still, plans can and often do take a conditional form. Just like I could say: “later, I am going to start a family”, I could say: “if I find a caring partner, I am going to start a family”. That my plan to start a family is conditional on my finding a partner does not make it any less of a plan, and it does not make my plan irrational in any way. In order to make the plan to start a family, then, I do not need to believe that I will find a partner – that is, I do not need belief-security of partnership. I do, however, need to *not* believe that I will *not* find a partner, otherwise my plan to start a family would indeed be means-end incoherent; irrational; fantastical. Similarly, in order to make complex rational plans for the future, the person who worries about her heart condition in Herington’s example does not need to believe that she will definitely live – that is, she does not need belief-security of her vital needs. She just needs to *not* believe that she will definitely die. In line with this, Bratman (1999) writes that “there need be *no* irrationality in intending to A and yet still not believing one will”, but “there *will* normally be irrationality in intending to A and believing one will *not* A” (p. 38, emphasis mine). Thus we do not need belief-security in order to function as rational planners.

Nonetheless, belief-security can contribute to human flourishing in a different way. In their study of “disadvantage”, Wolff and De-Shalit (2007) describe several manners in which people are *inhibited* in their flourishing when their functionings are or become *in*secure. When a person believes her functionings to be insecure, they point out, she tends to take actions to evade or brace herself for the blow, and these actions are often costly in themselves. They mention the example of someone who “fears being attacked on the street, and so has insecure bodily integrity”, and therefore chooses “always to travel by taxi, and suffer the financial costs, or simply not go out, and lose many opportunities as a result” (p. 68). If a person has security of a good, on the other hand, she does not need to spend her energy, time, and money on preventing or preparing herself for scenarios in which she does not enjoy the good. Therefore, she can achieve more well-being in the present. At the same time, she can make sure she is all set up to derive the maximum benefit from the good that awaits her. For instance, if someone anticipates that she will get a job in a different part of the country, she can arrange a nice place to live there at an early stage and thus get a head start when she takes up her new position. As a result, she can achieve more well-being in the future as well. Thus we can see that security can deliver an efficiency benefit that helps one to live a more flourishing life both in the present and in the future.

Although it is belief-security that does the main work here – it is because a person *believes* that she will enjoy a good that she forgoes costly precautionary measures now, and sets herself up for full enjoyment of the good later – this benefit will only fully obtain if belief-security is backed up by fact-security. If someone is in fact bound to enjoy some good, then the belief that she will enjoy it can help her to pursue well-being in an efficient way. But if she is *not* bound to enjoy this good, then she better prepare for the scenario that she not enjoy it. In that case, allocating resources to this scenario may not be a waste at all, but may actually be necessary for doing well in the future. Suppose someone believes that she will always enjoy perfect health, and therefore does not purchase any health insurance. If, against her optimistic expectations, at some point she does fall ill, and then cannot afford the costs of treatment, these expectations did not make her better off after all. In other words, the belief that we will enjoy a good yields a full efficiency benefit only if this belief is true.^11^

There is an additional reason why we might think that human flourishing is better served by belief-security if it is complemented with fact-security, which has to do with the particular character of this understanding of well-being. As noted before, human flourishing is not a purely subjective notion of the good: on this understanding, what matters for someone’s well-being is not only whether she *experiences* the world in a certain way – she *thinks* she makes a friend or writes a great novel – but also whether that experience is *real* – the friendship is *in fact* mutual and the novel is *actually* great. In line with this, we might say that it matters whether the *belief* that one will enjoy a good is also *true*. Not just because the efficiency benefit of belief-security can only fully be reaped when fact-security obtains as well, but also because a true belief has more intrinsic value than an untrue one.^12^ This line of reasoning is plausible: I think many would consider someone who truthfully believes that she will later have, say, good health, to be better off currently than someone who falsely believes so, even if neither of them acts on their belief in a way that delivers her an efficiency benefit. But, presumably, not everyone will agree on this last point. Still, my earlier point should be uncontroversial: belief-security, when backed up by fact-security, can help people flourish because it can help them prevent wasting resources in the present and get the most out of the good that awaits them in the future.

Thirdly, consider emotion-security, being the absence of fear. Obviously, fear is generally experienced as a negative emotion. Think of the scare we sense when hearing suspicious noises at night, the dread we feel at the thought of losing a sick family member, or the anxiety we experience in a panic attack. Fear can also make us suffer in a more indirect way: it can constrain our enjoyment of the physical and psychological capacities we have as human beings. Wolff and De-Shalit (2007) point out that stress and anxiety may hamper people’s bodily as well as mental health, and their ability to play and to plan (pp. 68–69). Nussbaum (2006) suggests that fear and anxiety can stand in the way of people’s emotional development (p. 77). Probably, many of us can relate to this on a personal level as well. Feelings of insecurity can gravely inhibit us in our normal functioning, by causing us difficulties to sleep, concentrate, and relax. Thus it seems that emotion-security serves a crucial function in our flourishing – not so much as itself a source of pleasure, but rather as a condition supporting various aspects of well-being.

Whether emotion-security, like belief-security, needs to be backed up by fact-security in order to make its full contribution to our well-being, is a difficult question. On the one hand, it could be argued that it is good not to suffer from fear in the ways specified above even if harm is in fact impending. Even if, say, we are about to be robbed of our wallet, perhaps it is good for us not to be afraid before it happens: because fear is unpleasant in itself, and because feeling frightful might actually undermine our ability to function properly both before and during the robbery (Herington 2019, p. 187). On the other hand, there are reasons to think that emotion-security is only good for us if it fits the facts we find ourselves in. For a start, it seems that feeling fear can have a function in making our mind and body alert to dangers, which in turn enables us to react to them timely and appropriately when they materialise. Not feeling fear may therefore work out badly for us if we do in fact have a reason to be fearful. In the above example, having a false sense of security might actually inhibit us from running away fast enough or screaming loud enough when the robber appears. This is in line with Aristotle’s position that it is important for emotions to be “appropriate”. Whereas the Stoics considered emotions such as fear and anger always inappropriate, Aristotle believed, as Kraut (2018) puts it, “not simply that these common passions are sometimes appropriate, but that it is essential that every human being learn how to master them and experience them in the right way at the right times” (section 5.1).

One additional reason for thinking that emotion-security better be backed up by fact-security takes us back once more to the particular character of human flourishing as an understanding of well-being. Remember that on this view, it is not only experience that determines our good, but also the match between experience and reality. A friendship that is *truly* mutual has more value than a friendship that only *appears* mutual to a person. Similarly, we might say that a rightful sense of security is intrinsically more valuable than a false sense of security. Again, I suspect that some people will find this intuitive and others will not. However, the general point that emotion-security contributes to our flourishing as a supportive condition for many of our pleasures and functionings should not be controversial.

Before closing, let me add one more point.^13^ This paper analyses the ways in which people’s security impacts their well-being without differentiating between the various goods that they can have (or lack) security of, or the various sources of their (in)security. But there is good reason to think that these factors do mediate the ways in which security affects well-being. People’s security of basic needs fulfilment probably makes a greater contribution to their flourishing than their security of less vital goods. And insecurity that is experienced structurally – think of the higher risk of violence faced by members of discriminated groups – is likely to reduce people’s flourishing more than insecurity that is rather incidental – think of the unease felt by someone who finds himself in a dark alley just once. These issues deserve more attention than they can be given here. One account that illuminates both of them is that of Wolfendale (2017). She proposes to incorporate into the right to security not only the usual requirement of basic physical safety, but also a demand for “moral security”: that everyone *believes* that one’s basic interests and welfare will be accorded moral recognition by society, and that society *actually* regards everyone’s interests and welfare as morally important (p. 238). Presumably, there exists not only a case for including this particular type of security among our basic human rights, but also for taking it to be of special importance for human flourishing.

All in all, we may safely conclude that security can make significant contributions to human flourishing. Fact-security generally means good news for our flourishing in the future; belief-security enables us to be more efficient in our pursuit of well-being; emotion-security supports our enjoyment of many of our human capacities. Furthermore, we can conclude that it matters a great deal whether or not the different aspects of security are aligned with one another. Firstly, if a person has fact-security of a good without belief-security and emotion-security, strictly speaking this does itself not even contribute to her current well-being. Secondly, if a person has belief-security without fact-security, this can actually stand in the way of efficient preparation for the future. Finally, if a person has emotion-security without fact-security, this may indeed detract from the sharpness she might need in order to deal with an impending harm. When it comes to its contribution to our flourishing, therefore, the comprehensive ideal of security as sureness turns out to be more than the sum of its parts.

## What is bad about security?

Might there also be ways in which it is not good for a person to have security of a good? Let me now explore for each of the three aspects of security whether they can also hamper human flourishing.

Firstly, consider fact-security again. Can it ever be bad for a person if she is bound to enjoy a good? At first sight, this seems an absurd question. We already learned that, strictly speaking, fact-security does not itself make a contribution to well-being. Instead, the contribution comes from the good security of which is at issue. By calling this thing a “good”, we assumed that it is something that contributes to this person’s well-being, in some way, at some point, either as a component of or an instrument for her flourishing. Therefore, it seems that, per definition, fact-security of a good cannot be bad for someone. On closer inspection, however, there actually is a way in which it can turn out badly for someone if she is bound to enjoy a good. This is when the good at issue might be good for her at a certain moment in time, but will be bad for her later.

Consider the following example. During my time as a student, I lived in a house together with twelve other students, with whom I had dinner every night, drinks every week, and a party every month. It was great fun, and I cannot imagine any other living situation that would have contributed more to my flourishing back then. Yet, it would not be good for me to still be living at that place right now. Indeed, it would have been bad for me if I had been bound to live there after I graduated. For one thing, having my own apartment is a far better fit with my present occupations, and communal living would actually inhibit my well-being presently. For another thing, I consider my life enriched by having experienced different living situations, and I would not have achieved such diversity in my life experience if I were still living in my student flatshare. The general point is this: something that is good for a person at some point need not be good for her forever. Therefore, we could say that fact-security of a good can, paradoxically, turn out badly for a person – although again, it must be noted that the negative effect on her well-being actually comes from the good in question, and only occurs as soon as this good stays or becomes present in her life when it is no longer good for her. Against this view one might raise two points, which are semantic but important.

For a start, it may be said that even if a person has fact-security of a good, she can still opt not to enjoy this good, and therefore it seems that fact-security cannot inhibit flourishing. Applied to the above example, the thought is that my fact-security of my flatshare would not prevent me from deciding to move out. However, as long as it is true that I have fact-security of my house, it must necessarily be the case that I have not (yet) decided to move out – for if I had decided to move out, I would no longer be bound to continue living there, and I would thereby have cancelled my fact-security of the house. Only if the good in question were the *option* of living in that house, would it be true that my fact-security of this good would not exclude the possibility of me moving out.

Relatedly, one might say that we should be stricter in our use of the word “good”. Arguably, the word “good” should be reserved for things that are good for people *at the point that they have them*. In the example mentioned above, this would mean that my flatshare would simply lose its status of “good” for me when what was at issue was my potential enjoyment of the house after my graduation; perhaps this status would then be passed on to the *option* for me to live there. Of course, we could agree to use the word “good” only in this stricter sense, and thereby make it per definition impossible for fact-security of a good to turn out badly for a person. The basic point, however, still holds true: fact-security of things that contribute to a person’s well-being at one point in time can indeed be disadvantageous for this person at a later point in time.

Secondly, let us revisit belief-security. Can it ever be bad for a person to believe that she will enjoy a good? One type of scenario was already discussed in the previous section: one believes one will enjoy a good, but unrightfully so. We saw that this could indeed be bad for a person because it could hinder her in preparing herself for what will come. Here I focus on scenarios in which a person believes she will enjoy a good, and this is actually true. Can that ever be bad? My thesis is that it can. Go back to the moment when you found out you got accepted to graduate school, or got hired at your job. Would that moment have been just as valuable to you if you were already sure of the happy news beforehand? I think most of us would answer “no”. Now remember a moment in your life when you were expecting one thing to come your way, but what you got was something completely different, which actually turned out great for you – perhaps a new hobby or haircut that was very different from what you planned for. Would you have preferred to have expected it beforehand? Likely the answer again is “no”. Larmore (1999) captures these intuitions in his insight that “being surprised by a good of which we had no inkling is itself an invaluable element of what makes life worth living” (p. 99). In a sense, living your life is like reading a book: a big part of the joy lies in not foreseeing what will happen next. Indeed, if the whole plot develops exactly according to your expectations, you might not even consider the story worth reading. For Larmore, this is one of the reasons to oppose an idea found in western political philosophy from Socrates to Rawls, namely that the good life is a life lived according to a rational plan. In the present context, it is a reason to think that belief-security can indeed sometimes impede human flourishing.

Thirdly, we go back to emotion-security. Can it ever be bad for a person to be without fear? We already learned that emotion-security might be bad when it is not accompanied by fact-security – in other words, when someone has a false sense of security. Again, let us put such cases aside and think for now of a person who is rightfully free from fear. My thesis is that even this can sometimes be bad. Of course, fear is mostly experienced as an unpleasant sensation. Yet, sometimes fear can be enjoyable as well. People with risky hobbies such as climbing mountains or lighting fireworks will readily confirm this. But also those who do not actively seek risks will admit that a certain level of fear can be pleasurable in some circumstances – when you pick up the phone to hear the result from a job interview, or right before you profess your love to someone. And one need not be a hedonist to acknowledge the value of such feelings: as noted before, pleasure may well be part of what constitutes a flourishing human life, even though such a life requires more than pleasure alone. I conclude that, by robbing us of enjoyable fears, emotion-security can indeed sometimes hamper our flourishing.

One final point should be noted.^14^ Aside from the specific ways in which security can hamper a person’s well-being analysed above, an abundance of sureness along any or all three of its dimensions might also inhibit human flourishing in a more general manner. This has to do with the fact that a good life is not the same as a life that is continuously smooth, easy, and painless. Facing and overcoming difficulty may indeed be crucial for developing the practical and rational abilities that we need in order to assume some kind of agency over our lives, and may well be essential to the good of achievement (see Bradford 2015). Insecurity – be it along the factual dimension, the belief dimension, or the emotional dimension – could be a source or manifestation of such difficulty, opening up chances for cultivating resilience and individual growing. Absolute and permanent security, on the other hand, may indeed obstruct these elements of flourishing.

All in all, we can conclude that security is not always and only good for people. Indeed, fact-security of a good can turn out badly for a person, and belief-security and emotion-security can inhibit her flourishing too – even if they are backed up by fact-security. These findings are far from trivial. They teach us that it would not be wise to “lock ourselves in” by securing forever what is good for us now, leaving nothing to be surprised by, nothing to be scared of. Also, there is good reason to think that, even if we wanted to create such complete security, we could not achieve it anyway. Some philosophers have argued that, however much we would like to foresee or control our future, many of the good things in life simply cannot be predicted or managed like that. Paul (2014), for one, points out that for a lot of big life choices we cannot know beforehand how they will turn out for us in the end – think, for instance, of the decision to have children. Nussbaum (2001), furthermore, draws attention to the elements of life that seem indispensable for the goodness of it, but are not entirely under the control of the agent living it – examples here include love and friendship with others. Although these thinkers focus on the lack of *control* we have with respect to matters such as these, it may also be argued that it is impossible for human beings to have *security* in the areas at issue. However, such an argument should be distinguished from the thesis defended here: regardless of whether it is *possible* for people to have security of goods, it is sometimes *not good* for them to have it.

## Conclusion

This analysis has shown that there are multiple ways in which security can contribute to our well-being: if we are in fact bound to enjoy a good, in principle this is advantageous for our flourishing in the future; if we also believe that we will enjoy this good, we can be more efficient in the pursuit of our well-being; if we also feel secure, this supports our enjoyment of our physical and mental capacities. For some of these benefits to obtain fully, however, it is important that our beliefs and feelings align with the facts. Furthermore, mirroring the upsides of security, there are also ways in which security can impact our flourishing negatively on occasion: it can stand in the way of the change, surprise, and pleasurable fear that are sometimes required for a good life too.

So what does a flourishing human life look like in terms of security? This research suggests that it has neither too little, nor too much security. There is a balance to be struck. Probably, the ideal level of security differs per person. Some people are like Hobbes, who claimed to have been born with fear as his twin (Hobbes 1680, p. 2), and took “the object of mans desire” to be “not to enjoy once onely, and for one instant of time; but to assure for ever, the way of his future desire” (Hobbes 1996, p. 70). Others are more like Nietzsche, who insisted that “the secret to harvesting the greatest fruitfulness and the greatest enjoyment from existence is to *live dangerously*” (Nietzsche 2018, p. 283). Presumably, in order to live your best possible life, you need a little bit of both.

Does this analysis also have political implications? More specifically, does it imply that the state ought to create particular forms of security for its citizens? Not necessarily. For one thing, making such an argument would require a study not just into the ways in which security itself affects people’s well-being, but also into the question what particular goods it would be best for people to have security *of*, and what sources it would be best for them to derive security *from*. For another thing, if such an analysis is supposed to inform the politics of contemporary liberal democracies, then the objectivist – or perfectionist – theory of well-being adopted here may not be the ideal place to start. The whole point of the liberal state, after all, is to allow for a diversity of subjective conceptions of well-being to coexist in society, and let citizens decide for themselves which particular idea of the good they want to pursue. The finding that certain kinds of security make people better off from the perspective of one specific idea of well-being would thus not suffice to show that the state should strive to provide that security for all.

An additional point that has to be noted here is that striving for security by the state is often not without its costs and dangers. As many scholars have pointed out (see, for example, Neocleous 2007; Waldron 2003), and as has become clear for instance from the ways in which governments have responded to the threats of terrorism and the coronavirus pandemic, the state’s aiming for security in practice often comes at the cost of people’s freedom. And the restrictions of civil liberties that may form part of the state’s security policies may in turn end up undermining citizens’ security. Finally, when considering if we should ask the state to relieve us from nagging insecurities, we should remember that a world with only absolute securities would not be good for us either. As this study has argued, a flourishing human life is also a diverse life, with occasional plot twists and a decent portion of suspense – be it with a solid foundation of security to build on.

## Footnotes

1 In addition to the works mentioned here, which are especially relevant for an inquiry about security and well-being, there are a number of writings in political philosophy that address security in relation to other topics, such as human rights (Binder and Binder 2019; Shue 1980; Wolfendale 2017) and emergency politics (Floyd 2019; Neocleous 2007).

2 By saying that a person is “bound to” enjoy a good, I mean that an all-knowing observer, aware of all the relevant facts of the world at present, would predict that this person will enjoy this good in the future. Borrowing the terminology of John (2011), I could say that a person has fact-security of a good if and only if, or to the extent that, “there is a warrant for her to believe” that she will enjoy this good “across the range of plausible futures” and “this belief would be true” (p. 73).

3 Admittedly, objective probability is a tricky concept (see Hayenhjelm and Wolff 2011; Perry 2007). Causal determinists would say that the only possible objective probability values are 0 and 1 (Schaffer 2007). Nonetheless, I follow Herington (2019) in assuming that whether a person is in fact bound to enjoy a good can indeed be viewed as a matter of (objective) probability.

4 Of course, we often do not have clear-cut beliefs about the probability that we will enjoy goods. Nevertheless, I follow Herington (2019) in thinking that we can indeed describe our credence that we will enjoy goods in terms of (subjective) probability.

5 For a plea for continuity, i.e. preserving old goods instead of replacing them by new ones, see Cohen (2011).

6 For an argument that does link (income) security to unconditionality, see Standing (2008).

7 For an analysis of the balance between luck and control in the good life, see Nussbaum (2001).

8 For a thorough account of the concept of certainty, see Reed (2008).

9 For an extensive treatment of robustness and its relation to the good, see Pettit (2015). The difference between security as it is understood here, and robustness as Pettit sees it, is also important for his republican theory of freedom. On this view, a person is free insofar as she enjoys non-interference across different possible *worlds* – that is, to the extent that her non-interference is *robust* (p. 3). This need not be the same as the degree to which she enjoys non-interference across different possible *futures* – that is, the extent to which her non-interference is *secure*.

10 This view has been contested by John (2011), who argues that physical security, objectively defined, is indeed a constituent of well-being. However, this argument has convincingly been refuted by Herington (2019, pp. 194-198).

11 It must be noted that the belief that one will enjoy a good *can* also yield an efficiency benefit if it is *not* true. This is the case when the costs one is bound to face when one does not anticipate some harm (say, the costs of medical treatment) are less than the costs of the precautions one would have taken had one anticipated this harm (the costs of health insurance). However, the efficiency benefit will of course be higher if one is bound not to suffer this harm at all. I therefore say that belief-security’s efficiency benefit only obtains *fully* if belief-security is backed up by fact-security.

12 If a belief is not only true but also justified, then the belief counts as knowledge, and the argument for its intrinsic value becomes even stronger. Here, however, I leave aside the question whether a belief is justified or not, because for the match between belief-security and fact-security to obtain, it is sufficient that a belief is true.

13 I thank one of the anonymous reviewers for raising this point and coming up with the example mentioned here.

14 Again, I thank one of the anonymous reviewers for making me aware of this point.
